# Vertebrate Genome Evolution in the Light of Fish Cytogenomics and rDNAomics

**DOI:** 10.3390/genes9020096

**Published:** 2018-02-14

**Authors:** Radka Symonová, W. Mike Howell

**Affiliations:** 1 Faculty of Science, Department of Biology, University of Hradec Králové, 500 03 Hradec Králové, Czech Republic; 2 Department of Biological and Environmental Sciences, Samford University, Birmingham, AL 35229, USA; wmhowell@samford.edu

**Keywords:** fish cytogenomics, repetitive sequences, rDNAome, genome evolution, AT/GC compositional evolution, quantitative cytogenomics.

## Abstract

To understand the cytogenomic evolution of vertebrates, we must first unravel the complex genomes of fishes, which were the first vertebrates to evolve and were ancestors to all other vertebrates. We must not forget the immense time span during which the fish genomes had to evolve. Fish cytogenomics is endowed with unique features which offer irreplaceable insights into the evolution of the vertebrate genome. Due to the general DNA base compositional homogeneity of fish genomes, fish cytogenomics is largely based on mapping DNA repeats that still represent serious obstacles in genome sequencing and assembling, even in model species. Localization of repeats on chromosomes of hundreds of fish species and populations originating from diversified environments have revealed the biological importance of this genomic fraction. Ribosomal genes (rDNA) belong to the most informative repeats and in fish, they are subject to a more relaxed regulation than in higher vertebrates. This can result in formation of a literal ‘rDNAome’ consisting of more than 20,000 copies with their high proportion employed in extra-coding functions. Because rDNA has high rates of transcription and recombination, it contributes to genome diversification and can form reproductive barrier. Our overall knowledge of fish cytogenomics grows rapidly by a continuously increasing number of fish genomes sequenced and by use of novel sequencing methods improving genome assembly. The recently revealed exceptional compositional heterogeneity in an ancient fish lineage (gars) sheds new light on the compositional genome evolution in vertebrates generally. We highlight the power of synergy of cytogenetics and genomics in fish cytogenomics, its potential to understand the complexity of genome evolution in vertebrates, which is also linked to clinical applications and the chromosomal backgrounds of speciation. We also summarize the current knowledge on fish cytogenomics and outline its main future avenues.

## 1. Introduction

### 1.1. Introduction into Cytogenomics

Cytogenomics, i.e., an integration of cytogenetic and genomic data and approaches supported by bioinformatics, is traditionally well established in clinical areas, particularly in cancer research and diagnostics [1,2]. Recently, cytogenomics has gained in importance in veterinary, e.g., [3] and in plant “-omics” research [4]. Along with progress in genomics, there was a literal call for “integrated cytogenomics” [5] in the recent endeavor towards the third-generation genome assemblies in avian genomics. In fish genome research, the first steps have already been taken [6,7,8]. Fish cytogenomics has been mentioned as the future pathway along which traditional fish cytogenetics should move [9] and is the logical outcome of the current integrating of fish biology research. 

What can this synergy between cytogenetics and genomics offer in groups of fishes with less explored and non-model genomes? What is the potential of cytogenomics in fish with their sometimes very large genomes consisting of numerous small chromosomes stuffed with repetitive sequences? What is the status quo and what might or what should be the future of fish cytogenomics? Do we at all need any cytogenomic research of basal vertebrates so different from mammals and we humans? Is there any potential practical use of detailed knowledge on fish genomes? We definitely sense a strong need for fish cytogenomics to develop and unfold itself further and to help us to understand the intricate fish genome evolution. Moreover, we also need fish cytogenomics to gain insights into the evolution of other vertebrate genomes, not only because all other vertebrates originate from a fish-like ancestor. Hence, fish genomes should be viewed as the first stage towards the even higher structural and functional complexity of avian and mammalian genomes. However, fish genomes are far less explored (particularly regarding their diversity), the novel methods are applied with a delay in fishes and therefore, there is lot of work to do to compensate this delay. Fish represent a diversified spectrum of ways of genome organization (Table 1) and mirror the complex genome evolution of all tetrapod vertebrates (Tetrapodomorpha) embedded within Sarcopterygian fishes, the sister branch of Actinopterygian fishes [10].

### 1.2. How Can This Review Been Utilized?

The goal of this review is to summarize existing data and approaches usable in downstream analyses of the genomic makeup of fishes and other vertebrates and to integrate them in contexts enabling a comprehensive understanding of the evolution of vertebrate genomes. In this way, we aim to provide a solid baseline for future fish as well as vertebrate cytogenomic evolution research showing the importance and advantages of work on fish chromosomes and genomes. This shall assist cytogeneticists to effectively utilize genomics resources. We outline the crucial and inevitable directions of fish cytogenetics and genomics as both fields of study move toward the new integrative cytogenomics with their databases growing daily. We also delineate and highlight the importance of the shift from the qualitative karyology and cytogenetics to the quantitative evolutionary-ecological and environmental cytogenomics. Hence, we have to start viewing the cytogenomic results in a broader, evolutionary and phylogenomic context and linking cytogenomic phenomena with their corresponding ecological and physiological causes and consequences. This review shall further serve as a reference to non-fish vertebrate genome researchers, where however, fishes are necessarily included as the most basal vertebrates representing the starting point of the vertebrate’s genome evolution. Finally, we present datasets of sequenced fish species listing their essential cytogenomic traits and information with potential for down-stream cytogenomic research and potential pitfalls and discrepancies to be regarded.

### 1.3. The Complex Evolution of Vertebrates Began with Fish

Understanding at least a basic framework and timescales of fish history substantially helps global understanding genome evolution in vertebrates. Jawless fishes as the first vertebrates with true bones arose in the early Paleozoic Era some 485 million years ago (mya). This crucial stage of our evolution remained conserved in lampreys and hagfishes that both considerably differ in their cytogenomic organizations from the rest of vertebrates (Table 1), although they show remarkable traits of convergent evolution with higher vertebrates (e.g., their adaptive immunity comparable with that of ours, [11]). However, lampreys and hagfishes differ also among each other, which is easily explained by their position on the phylogenetic tree, where five large extinct (†) groups are embedded between hagfishes and lampreys (although together known as cyclostomates):

Pteraspidiformes†, Anaspida†, Thelodontiformes†, Galeaspidiformes† and Cephalaspidiformes† [21,22]. It took another 65 myr for these fish to evolve jaws (chondrichthyans, ca. 420 mya). Here, we must be aware of the large gaps on the phylogenetic tree of *living* fishes, where other extinct lineages should have been otherwise placed (Placodermi†, Acanthodii†; [21,22]. After the jawed fishes appeared, it took another 169 myr to give rise to the ancestors of the living bowfin and gars (ca. 251 mya). However, both bowfin and gars are only the last survivors of much larger and very specious groups of once diversified and widespread lineages [23]. About 3 myr later, the ancestors of modern-day sturgeons and paddlefishes (Acipenseriformes) appeared (ca. 248 mya). The recent Acipenseriformes again represent only a small remnant group of once important radiations, whereas the order Paleonisciformes† went extinct completely without any survivors left [21,22]. The modern-day teleosts, which rule the world’s waters today, did not evolve until 225 mya. However, there was another completely extinct group embedded between Ginglymodi and teleosts, namely Pholidophoriformes†, making the phylogeny around to the origin of teleosts extremely difficult and so far unresolved (the Halecostomi-Holostei problems, [24]). This large gap among the surviving lineages is beside outstanding issues in their morphology reflected also in their cytogenomic organization [7,25] and illustrates the immense complexity and the current incongruence in the border(s) among Amiiformes (bowfin with its ancestors), Lepisosteiformes (gars) and teleosts. Originated in the marine realm, fishes radiated extensively in the sea [22] and colonized freshwater environments several times [26]. Immense selective pressures of these evolving environments must have shaped the genomes of untold thousands of fishes as they adapted to every conceivable ecological niche. Only recently, we have started realizing the extent to which the environment shaped their cytogenomic features [27,28] but we still have to fully understand them. This long time that elapsed during fish evolution reflects in the broad variety of ways that the organization of the fish genome has been structured and re-structured among cyclostomates, chondrichthyans, non-teleost actinopterygians, fish-like sarcopterygians and teleosts (Table 1). In parallel, a comparable variety in genome organization occurs within teleosts, although more than 50% of their species displays 2*n* = 48–50 [29]. This has to be regarded whenever any (cyto)genomic “generalizing” analyses are planned/performed. It is extremely simplifying to include merely 2–5 teleost species and consider them sufficiently representative of approximately 26,000 living teleost species, not including other Actinopterygia e.g., [30]. Unfortunately, there are frequent reductions of “fish” to teleosts (e.g., [31], etc.) leaving the entire potential of Actinopterygia described above unutilized.

## 2. The Importance of Fish Cytogenomic Research Demonstrated on Their Role in Medicine

Approximately 70% of the genes associated with human diseases have their functional homologs in teleosts [32,33], although teleosts diverged from lineages leading to humans more than 400 mya and underwent a teleost specific whole-genome duplication (WGD). On the other hand, the copy number of 5S and 45S rDNA is tightly regulated in mammals [34] whereas incomparably relaxed in fishes [8,35]. Copy number variation between 5S and 45S rDNAs in humans and mice can result in cancer [36]. In fishes, variations in a much higher extent can result in a speciation event as documented e.g., in *Coregonus* (Salmoniformes, [35]), in *Erythrinus* (Characiformes, [37]) and other numerous examples. This demonstrates the importance of fish cytogenomic research in medicine and in our understanding of vertebrates’ genome evolution. However, whereas the knowledge on mammalian (and yeast) rDNAs have already become established and currently receives its adequate attention with crucial findings being reported steadily. In fish, the research remains limited mostly to FISH (fluorescence in situ hybridization) detections of rDNA on chromosomes (for a survey see [38] or [39]) and to only several detailed molecular analyses of rDNAs in salmonids [40,41] and cichlids [42]. 

### 2.1. Why Is the Zebrafish the Most Important Fish Model?

Zebrafish (*Danio rerio*) have played an important role in accelerating our knowledge in embryonic development, regeneration, gene expression, transgenesis, environmental monitoring, drug discovery, cardiovascular diseases, immunology, infectious diseases, RNA splicing, stem cell biology and a host of other areas of importance to medical science [33,43,44,45,46]. At least 20% of the zebrafish’s duplicated gene pairs have been retained from this WGD [47,48,49,50]. This often causes altered gene expression and protein functions, such that the complement of the expression domains of both fish paralogs are equivalent to the single orthologue in other vertebrates. Despite this WGD, zebrafish and humans have about the same number of chromosomes and zebrafish chromosomes are mosaically orthologous to several human chromosomes [50]. Cancer researchers use the zebrafish model to study vertebrate gene function [51] since its embryos are virtually transparent. This has led to much knowledge in gene function and genetic diseases [52,53,54]. Zebrafish have been used to make several transgenic models of cancer (melanoma, leukemia, pancreatic cancer and hepatocellular carcinoma; [44]). Zebrafish express mutated forms of either the *BRAF* (B-Raf) or *NRAS* (Neuroblastoma RAS) oncogenes and develop melanoma when placed onto a p53 deficient background. These tumors strongly resemble human melanoma. The BRAF melanoma model was used to understand the function of genes known to be overexpressed or amplified in human melanoma [51,55]. One gene, histone methyltransferase called *SETDB1*, markedly accelerated tumor formation in the zebrafish, demonstrating its role as a new melanoma oncogene. *SETDB1* is further known to be involved in the epigenetic regulation central to tumorigenesis [51]. 

### 2.2. Other Fish Species Used as Medical Models Have Genomes Suited for Specific Human Diseases

The Japanese Medaka (*Oryzias latipes*) has a gene sharing over 95% identity with the human *HRAS* gene, which is one of the most frequently mutated genes in cancers [45]. The medaka is a complementary model to the zebrafish as it has many of its desirable traits [56]. Among Antarctic notothenioids, the Antarctic rockcod (*Notothenia corilceps*) has an extra stout mineralized skeleton. Some of these fishes demineralize their bone to increase their buoyancy to the extent that they develop osteopenia, a bone loss very similar to osteoporosis. Certain cichlids serve as models of craniofacial developmental disorders to predict and treat human craniofacial disorders [57]. They have evolved very different craniofacial morphologies dependent upon the diet to which the particular species has specialized and genes responsible for their craniofacial adaptations have been discovered. The blind cavefish (*Astyanax mexicanus*) has both surface populations living in the light and cave populations living in total or near-total darkness with retinal degeneration and albinism [45,58]. Some studies have suggested that certain eye development genes (*Paired Box 6* (*PAX6*), *sonic hedgehog*) are linked to eye degeneration [59]. Further questions are being currently solved since the full genome sequence of *Astyanax* is available [58] to determine the affected genes and elucidate their role in eye development [45]. Fair skin in fish and humans is predisposed to skin cancer. Also, the absence of melanin in the retina of cavefish may cause vision disease via often mutated gene OCA2 causing oculocutaneous albinism type II in humans [59] and is proof that the mutations in the same gene can result in the same phenotype in fish and humans [45]. Many killifishes (Cyprinodontiformes) make excellent models for aging research since they have adapted to life in the extreme conditions in the wet-dry savannahs of Africa. Eggs and embryos survive dry periods by undergoing diapause in the dry lake beds. When the rains come, they hatch quickly, and their life history is completed in the few months before the dry season arrives again. They have a very short lifespan even in aquaria under optimal conditions. Certain strains of this fish live only 10 weeks while others live 31 weeks. The fast aging in killifish show many of the same signs of older organisms, such as decreased fertility, cognitive decline, age-related molecular markers and high morbidity [60]. In the turquoise killifish, *Nothobranchius furzeri* aging is linked to an increase in cancer, infectious diseases, neurodegenerative diseases and circulation problems, hence this model is a useful model to study the aging process [60]. Toadfish (Batrachoididae) protects itself from predators by releasing urea to mask its scent and hiding spot and has a unique nitrogen excretion which makes them resistant to ammonia. Such a situation in the external or internal environment of humans would be harmful. The plain midshipman, *Porichthys notatus*, is a model for a human hepatic (portosystemic) encephalopathy, which is due to liver failure or excessive amount of nitrogen after kidney failure. The toadfish also serves as a model to study human sickle cell anemia and erythrocyte sickling under anoxic conditions. Under low oxygen levels, the toadfish’s erythrocyte sickles similar to the human mutant, malfunctioning hemoglobin known as HbS [61]. Swordtails and platyfishes are one of the oldest animal models for cancer research. It was also the first to present evidence that cancer has a genetic basis. Certain hybrids of platyfish (*Xiphophorus maculatus*) and swordtails (*X. hellerii*) develop malignant melanoma. The genetics of the tumor formation is complex and has been well documented [62]. The *Xiphophorus* model has been widely studied in an effort to better understand the mechanism of melanoma formation in humans. The *Xiphophorus* model is well established as its three genomes has been sequenced ([63] and Appendix A). Eels (Anguilliformes) are excellent models of bone demineralization and childhood kidney cancer (Wilms’ tumor). Physiological stress, fasting, or extensive migrations in eels cause bone resorption. Thyroid hormone has also been shown to be in involved in demineralization of their bone. An excess of the thyroid hormone can cause osteoporosis in humans, too. Wilms’ tumor occurs naturally in a high percentage of eels in nature [45] and in 1 in 10,000 children at an early age. Eels form a natural model which is not available elsewhere in order to study this childhood tumor. The bicolor damselfish *Stegastes partitus* develops multiple neurofibromas and pigment-cell tumors [64]. Rainbow Trout (*Oncorhynchus mykiss*) is one of the oldest models for studying human liver cancer (hepatoma) since it is particularly susceptible to environmental carcinogens, especially aflatoxin B1 produced by *Aspergillus*. The hepatoma in trout is strikingly similar in histopathology to that in humans. Mutations in the Ki-ras2 Kirsten rat sarcoma viral oncogene homolog (*KRAS*) oncogene often resulting in hepatoma are common in both trout and humans. The trout has since been used to identify other environmental carcinogens. Conversely, several chemoprevention studies have been done to determine dietary supplements that inhibit hepatoma in trout previously exposed to aflatoxin [65]. The mummichog (*Fundulus heteroclitus*, Cyprinodontiformes) has adapted to hypoxic water conditions, extreme high and low water temperatures, high and low salinity and has developed a tolerance to toxic pollutants produced by municipal, agricultural and industrial sources, as well as to oils and gasoline from fishing and pleasure boat traffic. Its ability to adapt to such conditions make it a model for the study of physiological resilience and adaptation [66] and to study human health concerns relative to the widely varying environmental insults that we encounter almost daily. 

### 2.3. Amazon Molly (Poecilia formosa) as a Cytogenomic and Epigenetic Model Species in Human Health Research

Amazon molly is a clonal species [67] and females do not undergo meiosis producing diploid eggs. When a male sperm from a related species stimulates the egg, it develops parthenogenetically—all offspring are clones genetically identical to their mother. This species has experienced first the classical cytogenetic phase of research (e.g., [68]), followed by genomics and (human) cancer research (oncogenomics) [66,67]. As a human disease model, the Amazon molly has been used in research into melanoma [69], infectious diseases [70] and thyroid cancer [71]. It is of interest that [69] introduced a microchromosome into Amazon molly genome inducing susceptibility to melanomas. The next stage was using zebrafish as a single gene knockout model in epigenetic cancer research. The histone demethylase, *KDM2A*, is thought to play a role in silencing transcription in humans. Otherwise, very little is known about its role in vivo in development and disease. Scahill et al. [72] discovered that the loss of the orthologous *kdm2aa* in zebrafish is disruptive to transcriptional processes and produces a high frequency of melanomas. The discovery of the *kdm2aa* mutants represents the first single gene knockout available for the study of melanoma induction. This zebrafish model is important as the World Health Organization reports 132,000 human melanoma skin cancers occur globally each year [72].

Fishes further represent important models also for environmental genomics [73] and for aquaculture genomics, genetics and breeding [74].

## 3. rDNAomics—Where Fish Ecological Cytogenomics Meets Human Cancer Genomics

**Figure 1:**
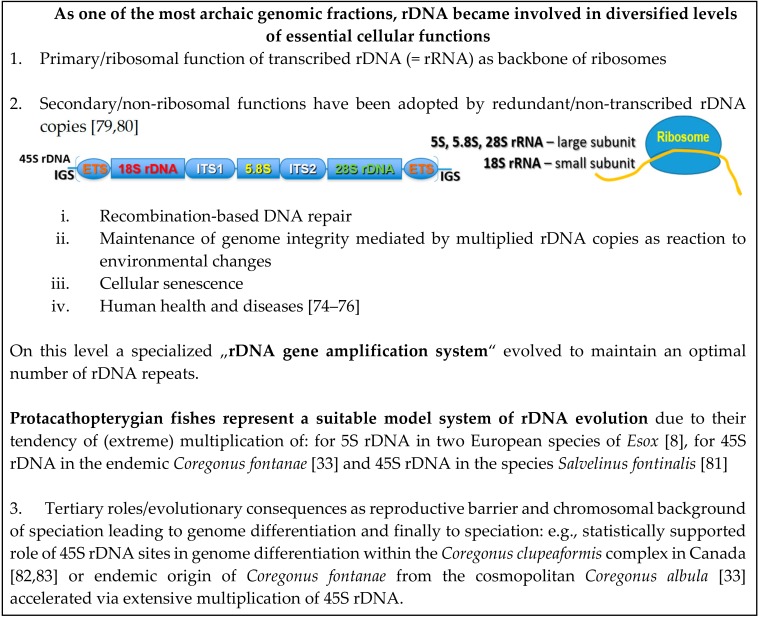
Three levels of ribosomal DNAs (rDNAs) functionality, generally and specifically in fishes.

Fish cytogenetics is largely based on chromosomal mapping DNA repeats that still represent serious obstacles in genome sequencing and assembling, even in model species. This has resulted in an immense amount of cytogenetic records including a still increasing number of “bursts” or “explosive spreading” of rDNA across chromosomes mostly in freshwater fishes. Only the availability of combined genomic data (e.g., Illumina and PacBio in the case of pikes, [8]), has enabled to quantify these extremely amplified rDNAs and to analyze them in a broader molecular context. At the moment, such data are available only for *Esox lucius* and they show that the copy number of the 5S rDNA fraction corresponds to the entire human gene number, i.e., about 20,000 5S rDNA copies. This means that in pike solely the 5S rDNA expanded to such an extent inconceivable in human or mouse genome and left the 45S rDNA fraction unamplified. Based on currently available cytogenetic data, similar situation in the 45S rDNA fraction can be expected in salmonids and in other fish groups (e.g., erythrinids, etc.) with the awaited increasing availability of hybrid sequencing. Then, we will be able to better analyze the causes and consequences of these phenomena. The results presented by the Animal rDNA Database [39] indicate that the freshwater environment might have favored these extreme amplifications of only one of the two rRNA gene fractions. This clearly rules out the primary ribosomal function of amplified rDNA molecules. Below in Figure 1 we summarize and visualize our current knowledge on coding and non-coding (non-ribosomal) functions and roles of rDNA generally and in the case of formation of reproductive barriers in fishes. The origin of these copy number bursts of rDNAs might be potentially related to the “rRNA gene amplification system,” which is finely tuned to maintain or, when necessary, to recover a particular and species-specific number of rDNA copies (explained by [75], section 10.4). The exact molecular mechanism(s) are the matter of vivid discussions and speculations at the moment (e.g., [8]) and include among others unequal sister chromatid recombination or retrotransposition. However, evidence accumulates that nucleolus (i.e., sites of the active rDNA transcription) and alone the rDNA copy number are involved in human diseases, cancer predisposition and other oncogenic activities related to genomic instability [76,77,78].

## 4. State-of-the-Art in Fish (Cyto)Genomics—The Starting Point

Several reviews on fish genomics have been published during 2003–2013 [77,78,84] and one recent (2016) special issue called ‘Fish, Genes and Genomes: Contributions to Ecology, Evolution and Management’ [85], which is mostly focused on population genetics and population and conservation genomics. In the meanwhile, numerous fish genomes have been published and several major milestones have occurred in the field of fish molecular cytogenetics: (1) Fish Karyotypes was published [86]—this immense work summarized previous knowledge and still provides a valuable reference overview of karyotypes and where available other karyological and cytogenetic traits in 3425 species/subspecies of extant jawless, cartilaginous, ray-finned and lobe-finned fishes and becomes thus an important reference tool; (2) A still continuing boom of FISH technique in fish resulting in the still increasing amount of molecular cytogenetic data; (3) Availability of more sophisticated and informative methods like GISH (genomic in situ hybridization) and CGH (comparative genomic hybridization) in fish resulting in comparative studies ([35,87,88] and more); (4) Large fish genomes with record chromosome numbers have been documented and analyzed and shifted the limits of fish genome size ([89] *Acipenser brevirostrum*, *A. mikadoi*, *Diptychus dipogon*). In parallel, the first non-human vertebrate and at the same time the first fish genome has been published—the *Takifuga rubripes *and two other pufferfish genomes thereafter (*T. flavidus *and *Tetraodon nigroviridis*) followed by newer versions of their genome assembly (details below). These fishes are of tremendous interest to fish cytogenomicists and generally to vertebrate genomics since they involve several crucial phenomena of the fish genome evolution that still represent outstanding questions. They have the smallest known of vertebrate genomes (350–500 Mb, [90]; Appendix A), which was the impetus for their sequencing. This among others enabled the first accurate prediction of the number of human protein coding genes [91]. However, their compact genomes retain similar chromosome numbers as teleosts with doubled genome size (i.e., 2n = 42/44), ([90]; Appendix A). Another notable feature of the tetraodontid genome its increased GC content—their GC-rich regions are gene-rich as in mammals [90], although the typical mammalian type of chromosome banding does not occur in pufferfishes [7]. On the other hand, there are other teleost species with comparably high genomic GC content (e.g., *Clupea, Gasterosteus, Gadus,* for details see Appendix A), however, without the extreme genome size reduction and genome compactness as in tetraodontids. A situation so far unprecedented among all fishes has been documented in extant gar genera (*Atractosteus* and *Lepisosteus*, Lepisosteiformes). Although their genomic GC content is not so increased as in pufferfishes, or as in other aforementioned species with unreduced genome size, their AT/GC compositional heterogeneity is unparalleled among cold-blooded vertebrates and is cytogenetically detectable in the same way as in mammals i.e., G-banding and AT/GC banding functionality [7]. This briefly illustrates the extent and the complexity of genome organization within fishes and the need to exploit the available resources in integrative cytogenomic approaches to at least partly clarify factors involved in the evolution of functional genome organization in vertebrates. 

## 5. Fish Cytogenomics—Practical Application of Integrated Cytogenetics and Genomics

### 5.1. Assignment of Linkage Groups to Specific Chromosomes Using FISH with Sequenced BACs

This approach has been so far applied only in several model fish species: zebrafish [92], rainbow trout [93], Atlantic salmon [94], Nile tilapia [95]. However, due to the workload required not all species have been processed in this way and even in species with genomes assembled to the chromosome level their linkage groups (LGs) still have not been assigned to chromosomes. Hence, there still exists need to continue in this effort in model as well as non-model species e.g., spotted gar [7,82], Lake Whitefish [83,96], Northern pike [97], etc. that will finally allow for down-stream analyses and combinations of data obtained by molecular cytogenetics and genome sequencing. This is crucial e.g., to explore the (1) genomic context of the rDNA sites on specific chromosomes; (2) the DNA sequence of centromeric and pericentromeric regions that frequently harbor diverse repetitive elements and show either AT- or GC-richness; finally, (3) identification of residual tetrasomic sites in paleo-tetraploids (salmonids) which is currently impossible by means of genomic and bioinformatics tools [98]. However, using FISH mapping of BACs, we have identified already two of eight predicted residually tetrasomic sites (i.e., sites preserving the ancestral tetraploid condition within the otherwise secondary diploidized genome) in the Lake Whitefish (*C. clupeaformis*, [99]). 

### 5.2. Quick Qualitative Analysis and Visualization of AT- vs. GC-Rich Chromosomal Regions

**Figure 2:**
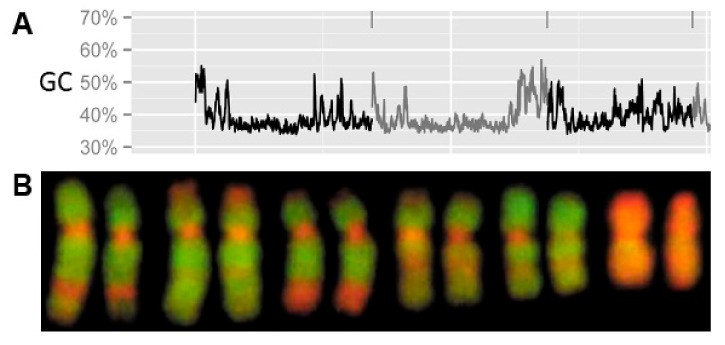
(**A**) GC-profiles across three linkage groups (LGs) so far unassigned to their corresponding chromosome pairs showing fluctuations in GC-percentage produced using the chromoplot tool. These three linkage LGs are arranged according to their numbers in the Ensembl [103] and separated by vertical lines above profiles and by the alternation of gray and black colors (*x*-axis—genome position, *y*-axis—GC%); (**B**) Partial karyotype stained with 4',6-diamidino-2-phenylindole/ Chromomycin A_3_ (DAPI/CMA_3_) showing six pairs of larger chromosomes of the spotted gar with altering GC-rich regions in red and AT-rich regions in green. Reproduced with permission from [7].

Quick qualitative analysis and visualization of AT vs. GC rich chromosomal regions utilizing fluorescent staining specific for AT (DAPI) and for GC (Chromomycin A_3_ (CMA_3_), 7-amino-actinomycin D (7-AAD), propidium iodide). This is a particularly useful approach in combination with bioinformatics analysis producing AT/GC profile across chromosomes/LGs hence quantifying cytogenetic data. Basically, there are two possible approaches producing AT/GC profiles across linkage group/chromosome: (1) chromoplot [7] calculates and plots the absolute GC percentage (Figure 2) or (2) the tool isoSegmenter [100] segmenting genome into pre-defined and broadly used concept of “isochores” (i.e., large genomic regions homogeneous in their GC content, *sensu* [101]). This DNA compositional cytogenomics gained in importance due to the recently uncovered AT/GC compositional heterogeneity in the ancient gars [7]. This finding means that it is crucial (a) to change our attitude to generally accepted compositionally homogenous fish genomes, which is not true anymore; (b) to revisit the so far obtained results and to employ e.g., the simultaneous DAPI/CMA_3_ staining together with attempts of G-banding in fishes. Some authors tend to present DAPI stained metaphases separately from the CMA_3_ stained ones or not to show one of them. This makes the situation complicated for any serious reason and does not allow for proper exploitation of the data (i.e., a part of information would be missing despite its actual availability); (c) this sheds new light on the vertebrate genomic DNA composition generally because so far the broadly accepted concept has considered fish and amphibians AT/GC homogenous whereas only birds and mammals were AT/GC heterogeneous, with transient situations in reptiles [101]. However, the bromodeoxyuridine (BrdU)/pulse replication labeling does produce reproducible bands in both compositionally heterogeneous higher vertebrates and homogenous fish and amphibians (e.g., for fish [102]). Therefore, in the light of findings in gars and the bowfin, it would be highly desirable to perform a large-scale comparative cytogenomic study across fishes to be able to exactly analyze the potential banding pattern in fishes so far considered compositionally homogenous. Namely, the BrdU banding pattern should provide a “scaffold” of expected bands, which should be then analyzed with DAPI/CMA_3_ and in parallel on the LG profiles. In this way, we should be able to quantify the cytogenetic thresholds of chromosomal band visualization and make the approach of chromosomal banding more sensitive to the putative less heterogeneous pattern in fish genomes. This step will be essential to properly understand the issue of compositional organization of fish and amphibian versus avian and mammalian genomes and finally the vertebrates’ genomes generally. The quantification on AT/GC profiles will require higher versions of better genome assemblies assembled to the chromosome level and with already filled gaps (or filled as much as possible). This means, that the so far available versions of genomes were not yet suitable for these analyses and hence, fish researchers have not yet missed anything. 

### 5.3. Cytogenetic Mapping of Repetitive Sequences on Conspecific Populations

Cytogenetic mapping of repetitive sequences on conspecific populations exploring their participation in evolutionary diversifications of different vertebrate species. In studies by [83,96], *population* molecular cytogenetics was shown to be an irreplaceable tool in exploring the population dynamics of rDNA sites. On the example of the Lake Whitefish (*Coregonus clupeaformis*, Salmonidae), we have documented that the differential dynamics of rDNAs across chromosomes participate in the evolutionary diversification of fish genomes. Similar studies performed in Erythrinidae (Characiformes) e.g., by [37,104] (this issue and citations therein) show comparably high intra-species evolutionary dynamics in rDNA and also other repetitive DNA in the genera *Erythrinus* and *Hoplias*. These and other similar finding originating from *freshwater* fish groups show the relevance of the FISH-based rDNA mapping (1) for understanding evolutionary mechanisms underlying ecological speciation [35] and (2) as an important tool how to tackle incipient and established biodiversity [105].

### 5.4. Cytogenomics of Duplicated Genomes—Understanding Mechanisms of Genome Evolution in Vertebrates Which Have Undergone Whole-Genome Duplication(s) 

Whereas there are no further WGD events in higher (warm-blooded) vertebrates, in lower vertebrates, particularly in fishes (recent review by [106]) but also in amphibians and to some extent in reptiles [107], there are numerous examples of WGDs. Hence, fish genomes offer irreplaceable insights into the diversity of evolutionary patterns in Sarcopterygia and Actinopterygia (Table 1). However, to sequence and above all to assemble such genomes remains challenging (bichirs, lungfishes, sturgeons, salamanders). Hence, the “classical” cytogenetics still represents an important tool to analyze these genomes and the post-WGD evolution [20,89,108]. Not only in these cases, cytogenetics can largely benefit from genomics and bioinformatics and compensate for the obstacles during sequencing and genome assembling ([19], this special issue).

## 6. Fish Cytogenetics and Cytogenomics in the Era of Digitalized Biology—The Boom of Databases

### 6.1. Databases for Fish Biology

Currently, fish genetics is beginning to benefit from increasing diversified efforts to build databases to compile and curate the increasing amount of molecular data on fish genomes. Hence, despite the devotion to fish genomics of the Indian subcontinent, we can explore and utilize in evolutionary studies e.g.: “FBIS: A regional DNA barcode archival and analysis system for Indian fishes” introduced by [109], “FMiR: A Curated Resource of Mitochondrial DNA Information for Fish” introduced by [110], “FishMicrosat: a microsatellite database of commercially important fishes and shellfishes of the Indian subcontinent” by [111]. Microsatellites of 31 fish species not confined to India are available in the microsatellite database MSDB [112]. The particularly important tool “Fish Karyome” is now available in its upgraded version [113]. Beside these four tools originating from India, the Animal rDNA database [39] including in its current version 546 fish and fish-like species is a highly relevant and informative tool usable in evolutionary-ecological fish cytogenomics or rDNAomics. Genome size of more than 2000 fish and fish-like species is currently available at the online database [12]. General data about fish biology, ecology and biogeography are already traditionally provided by Fishbase [114] and represent the first-choice tool for any evolutionary studies, including cytogenomics, in the ecological context. An online database specialized on B chromosomes and involving fishes is described in the Section 8.2 dealing specifically with B chromosomes.

### 6.2. History of the Zebrafish Reference Genome

History of the zebrafish reference genome started in 2001, when the Wellcome Trust Sanger Institute initiated the zebrafish genome-sequencing project and selected the Tübingen zebrafish reference strain as it had been widely used to identify mutations affecting embryogenesis. The Zv8 assembly was a hybrid of high-quality clone sequence (83%) and whole-genome shotgun (WGS) sequence (17%), with a total size of 1.412 gigabases (Gb). The clone and WGS sequence is tied to a high-resolution, high-density meiotic map called the Sanger AB Tübingen map (SATmap). This full genome sequence was made available to the public at the NCBI Zebrafish Genome Page and is maintained by the Genome Reference Consortium (FishMap Zv8 [115]). In 2009, the Institute of Genomics and Integrative Biology in India sequenced the genome of a wild zebrafish strain. The genome contained about 1.7 Gb and when compared to the Zv8 variations were found in over 5 million nucleotides and over 1.6 million indels. Later, the zebrafish reference genome was published, consisting of 1.4 Gb and over 26,000 protein-coding genes [32]. After the release of the Zv8 project, they joined the Genome Reference Consortium (GRC) for further improvement and maintenance. The GRC has now released a new reference assembly, GRCz11. Sanger’s GRC partners at the ZebraFish Information Network (ZFIN) continue in updates and maintenance. In parallel, Amores & Postlethwait [116] recognized a need for molecular cytogenetics in zebrafish and performed an exhausting study to facilitate the unambiguous cytogenetic identification of each individual chromosome. Several further molecular cytogenetic studies localized repetitive sequences in zebrafish [117,118,119].

### 6.3. Fish Genomes Available

Using the NCBI Genome Browser [120] and filtering for the Kingdom “Eukaryota,” Group “Animals” and Subgroup “Fishes” we find further about 90 fish species with genomes sequenced and assembled to the diverse levels. Additional fish species with a sequenced genome available can be found in a literature search resulting at the moment with about 95 species. These currently available species are listed in Appendix A, an online continuously updated version of this list will be available on the web page [121]. This list in alphabetical order includes: 2 lancelets, 1 hagfish, 2 lampreys, 1 shark, 1 ray, 1 gar representing the non-telesost Actinopterygia, more than 80 teleosts and 1 coelacanth of the group Sarcopterygia. In this dataset, we integrate basic genomic with basic cytogenomic traits—genome size in Mb based on sequencing, genome size as C-value originating from [12]; linkage groups (haploid) and diploid chromosome numbers; level and number of assemblies (i.e., draft, contig, scaffold, chromosome level), genomic GC percentage and sequencing platform applied emphasizing the PacBio technology with its particular relevance for chromosomal and repetitive DNA studies. The phylogenetic “coverage” of sequenced fish and fish-like species is provided in Appendix A and will be also online and continuously updated. Some of the model fish species have been (re)sequenced and (re)assembled up to 4, 5 times (see column #Assembly in Appendix A; details e.g., at [122] or at the Genome Assembly Database of the European Nucleotide Archive (ENA, [123]). Importantly, each new version means filling more gaps, improving scaffolds towards the chromosome level, improving of genome size estimations and proportion of repetitive sequences e.g., [124]. 

Sometimes substantially different results are found than in the previous genome versions (e.g., in Atlantic cod, [124]). Moreover, there are strong indications that numerous non-model genomes contain widespread and predictable assembly and annotation errors [30], which means that using their improved versions will be critical. Other de novo draft genome assemblies of 66 teleost species are available through [125] (these species will be included in further online version of Appendix A). Further, the Genome 10K Project aims to sequence and analyze 10,000 vertebrate species including fish and hence other fish genomes are expected to be available in the future [126]. Beside the aforementioned genomic sequences, 24 fish transcriptomes are available through the PhyloFish database [127]. On the top of that the Fish T1K platform announced transcriptomes of 124 actinopterygian fish species (covering 46 orders and 99 families) to have been already sequenced and further 59 species were in progress [128]. Since this web-based information has been updated only in 30 April 2015, the results might have been already substantially more progressed. Genomes assembled to the chromosomal level are particularly important for cytogenomics—currently there are 18 species (see Appendix A). Once the available linkage groups (LGs) are assigned to their chromosomes, the cytogenetic results will be directly applicable in genomic data and vice versa. This is however, still in the future, hopefully not too far away.

### 6.4. Future of Fish Cytogenomics = Phylogenomics

The abovementioned tools are together with the most recent fish phylogenetics published by [22] and [10], an excellent starting point for the application of modern phylogenetic comparative [129] and other quantitative methods exploring cytogenomic phenomena in the broader eco-evo context. 

## 7. Quantitative Eco-Evo Cytogenomics

Fishes are the only group of vertebrates, where relationships between genome size and essential cellular parameters, sometimes called cytogenomic ratio, remain uncertain [130]. In fish, similarly as in other vertebrates and eukaryotes generally, two opposite sets of theories attempt to explain the mechanisms behind the large variation in the amount of non-coding DNA—adaptive and non-adaptive theories [131,132,133]. Genome size is negatively correlated with GC percentage in several fishes, like *Tetraodon*, *Takifugu*, *Gasterosteus*, etc. (Appendix A). On the other hand, also larger fish genomes show GC increment (*Clupea*, *Gadus*) or even the mammalian like GC heterogeneity (gars, [7]). There is also a clear effect of environment and life-style on the GC content in teleosts [134]. Further, genome size obviously correlates with chromosome numbers, although considerable modifications in genome size can occur largely independent of changes in chromosome counts [29]. Here, the role of environment has been already repeatedly evidenced: occurrence of larger genomes in freshwater versus marine fishes [28,135,136], so far not recorded polyploidy in marine actinopterygians versus frequent incidence of polyploidy among chondrichthyans [12,15] and freshwater actinopterygians [28,29] and higher cytogenomic ratios in chondrosteans than in actinopterygians as in cold-water fishes relative to their warm-water counterparts [130]. These few examples show the complex network of numerous interactions on diverse levels ranging from molecular, over developmental, physiological and life-history traits to the environmental. 

However, we still need to better explore and understand all these levels and the interactions, since there are several major topics and related outstanding issues in fish quantitative eco-evo cytogenomics, where the genomics resources, e.g., data on exon/intron sizes and counts will be highly desirable to complement the existing robust datasets obtained from cytogenetics and flow cytometry: (1) the conundrum of remarkably constant diploid chromosome number (48–50) even among species that differ significantly in DNA content [28,135]—are there any phylogenetic constrains and/or selective pressures directing chromosomal evolution towards 2*n* = 48–50? In other words, do we have to look for potential nucleotypic limitations of genome size on cellular and organismal phenotypes? i.e., any links between rate of development, life-span length, metabolic rate, body size and cytogenomic parameters?; (2) the role of water environment (freshwater x marine) in the dynamics of evolution of genome size, chromosome number, fundamental number etc.—occurrence of larger genomes in freshwater versus marine fishes [28,135,136]; (3) generally the incidence of polyploidy only in the freshwater environment with the exception of chondrichthyans; (4) origin of the genomic gigantism (obesity) in lungfishes and bichirs [12,17,137].

Utilizing increasingly available karyological and cytogenetic data, several attempts have been performed to quantify these purely qualitative data and assess them in ecological, life history traits and physiological context. In this way, chromosome and chromosome arms (FN) numbers have been compared as a measure of chromosomal dynamics several times [138]. These studies indicate a chromosomal stability and conservatism in marine fishes although limited to restricted regions and groups [138]. All these studies represent important first steps although they are largely limited to a narrow subset of fish lineages, limited geographically and certainly not exploiting the available data and advanced cytogenomic methods. However, there is an essential shift from the previous mostly descriptive approach towards understanding the phenomena observed at the functional stage. 

## 8. Sex and B Chromosomes, Nuclear Architecture and Genome Evolution Research

Here we summarize three further aspects of genome evolution, where rDNA does play an important role and where the integrative cytogenomic already proved its potential. B chromosomes were among others proved to originate from sex chromosomes [139] or to interact with sex determination [140].

### 8.1. Sex Chromosomes in Fish

Sex chromosomes in fish are exhaustively discussed in another paper of this special issue and illustrated on the well explored example of Neotropical fishes [104]. We would like to highlight the contribution of rDNA regions in sex chromosomes evolution [104,141] and the cytogenomic approach that proved successful in combining both cytogenetic and genomic data e.g., [141] in the two most important model species. The mechanism of sex-determination in zebrafish is of importance to understand if it is to serve as a vertebrate model system to study human development and disease. However, past researchers have failed to find either an XY, ZW, multiple sex-determining mechanism, or environmental determination. Recently, using cytogenomics, this question has been mostly answered. A novel genetic map of single-nucleotide polymorphism (SNP) was used in a genome-wide linkage study of sex-determination in zebrafish [142]. Loci were identified on zebrafish chromosomes 5 and 16. Chromosome 5 locus contains *dmrt1*, a gene found in sex determination from fruit flies to humans. A mutation in the orthologue of this gene in humans results in complete sex reversal of XY individuals. Chromosome 16 contains *cyp21a2*. Mutation of the human orthologue of this gene is a common cause of pseudohermaphroditism. Recently, zebrafish chromosome 4 has been identified as a sex chromosome along with the sex-linked genes on chromosomes 5 and 6 discussed above [143]. In zebrafish, there is a combination of effects on the genome, germ cells and the environment with influences from epigenetic factors. However, the primary factors in sex-determination in zebrafish remain controversial [143]. The Japanese Medaka has an XX-XY chromosome-based sex determination similar to mammals with the male determining master regulator genes on the Y chromosome. Interestingly, this mode is not conserved even within the genus *Oryzias* [144].

### 8.2. B Chromosomes (Not Only) in Fish

B chromosomes (not only) in fish is another area of biology, where cytogenetics successfully and productively meets genomics and rDNAomics [145,146]. B chromosomes are known to contain rDNA frequently e.g., [147], summarized by [79,139]. B chromosomes have been identified e.g., in seven South American and in fourteen African cichlid species [143], in the genus *Poecilia* as we already mentioned by [148], in the bleak (*Alburnus alburnus,* Cyprinidae) [149] and in three species of thorny catfishes [150]. The complete list of B chromosomes identified in fishes is available in a specialized database ‘B chrom’ [151] by [152]. There are 278 entries are listed comprising approx. 120 species (depending on the species status and the level of species identification; accessed on 24 January 2018).

### 8.3. Nuclear Architecture

Nuclear architecture—nucleolus and rDNA emerged as important components of the nuclear architecture [78] and as indispensable components of mechanisms maintaining genomic integrity [81,153,154]. Moreover, the repetitive nature of rDNA and other repetitive genes (e.g., histones) results in a high evolutionary dynamics, known also as evolutionary hotspots [80,155]. Therefore, to understand the complex evolutionary structural as well as functional, mechanisms in vertebrate genomes, it is crucial to view the current cytogenomic knowledge also in the context of nuclear architecture, regulation of gene expression, role of transposons and epigenetics. At this stage, any attempts to explore structural aspects of interphase nuclei in fishes generally are missing. The single study on basic organization of two cold-blooded vertebrate genomes by [156] demonstrated that gene-rich regions in one amphibian (*Rana esculenta*) and one reptile (*Podarcis sicula*) occupy the more internal part of the nuclei, whereas the gene-poor regions occupy the periphery. This finding is similar to that previously reported in warm-blooded vertebrates, despite the lower GC levels of the gene-rich regions of cold-blooded vertebrates [156] and citations therein. In Atlantic cod, Kirubakaran et al. showed an example of putative directional selection for retaining two adjacent inversions on LG1 [157]. These inversions repress meiotic recombination in crosses. Moreover, the chromosomal block with these inversions harbors 763 genes, including candidates regulating swim bladder pressure, heme synthesis and skeletal muscle organization conferring adaptation to long-distance migrations and vertical movements down to large depths. Despite interbreeding between forms with (migratory ecotype) and without (stationary ecotype), the inversions are maintaining genetic differentiation [157]. 

## 9. Roots of (Population) rDNAomics in Fish Cytogenetics—rDNAomics as Another Dimension of Environmental Genomics 

**Figure 3:**
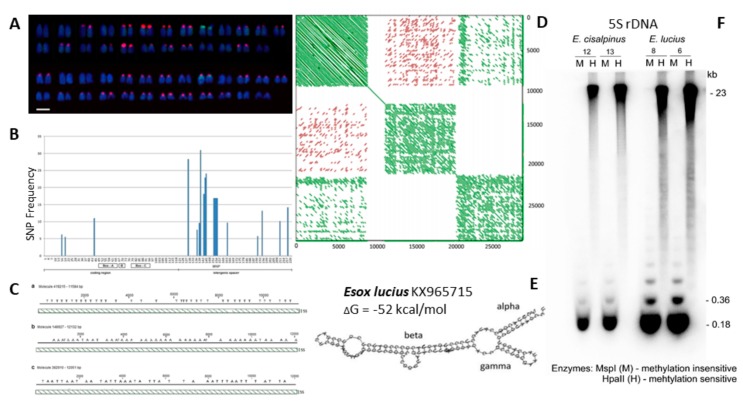
Summary of an integrative cytogenomic study on the rDNAome in European pikes (*Esox lucius* and* E. cisalpinus*). (**A**) Fluorescence *in situ* hybridization (FISH) with 5S rDNA (red) and 45S rDNA (green); (**B**) Distribution of single-nucleotide polymorphism (SNPs) along the *E. lucius* 5S rDNA unit obtained from Illumina reads showing absence of SNPs in the internal controlling region composed of Box-A, IE and Box-C elements; (**C**) Distribution of variants in intergenic spacer regions (IGS) in three PacBio reads (a–c). Slanted lines indicate tandemly arranged units visualized through the alignment of reads (*x*-axis) with a 5S gene (*y*-axis); (**D**) Higher-order organization of 5S rDNA arrays in *E. lucius*. Self-to-self comparison of long PacBio molecules representing three groups; (**E**) 5S rRNA domain reconstruction of *E. lucius* indicating its potential functionality; (**F**) Methylation analysis of 5S rDNA by the methylation-sensitive HpaII (H) restriction enzyme and its methylation-insensitive MspI (M) isoschizomere. Reproduced from [8].

There is a long tradition in chromosomal mapping of rDNA sites in the fish cytogenetics. This descriptive and qualitative work resulted in astonishing findings of extremely multiplied rDNAs, both 45S and 5S rDNA [8,35,37,158], for numerous other examples, see the animal rDNA database [39]. Mapping of rDNAs (but also of other highly repetitive DNA fractions as e.g., histones and transposons ([159,160]) across populations proved to be an important tool to explore the (sub)chromosomal background of populations’ diversification, incipient speciation and finally completed speciation events. These repetitive sequences appear to evolve at a higher rate and their mapping hence enables to catch various stages on the gradient of genome diversification, sometimes even stages that are not distinguishable on the morphological or genetic level ([161] vs. [35]). However, only in combination with other molecular methods and genomic data, a precise quantification and detailed insight became possible [8]. In this way, we documented a peculiar higher-order organization of the extremely amplified, potentially functional and massively methylated 5S rDNA in two species of European pikes, whereas the 45S rDNA fraction was ascertained in both of the pike species not to have undergone any amplification (Figure 3). 

Interestingly, Salmoniformes, the sister lineage of Esociformes, where pikes belong to, tend to amplify 45S rDNA with mostly stable and low 5S rDNA copy numbers [35]. Hence, this whole group of Protacanthopterygia (i.e., Salmoniformes and Esociformes, [20]) provides a suitable model system for further exploring and above all understanding the evolutionary dynamics of their rDNAome. Already the available findings in fish rDNAs are crucial since they show the immense differences between the genome and rDNAome complexity in lower and higher vertebrates. Namely, the discrepancy between copy number of 5S and 45S rDNA (e.g., [76]), which is being tolerated by fish and contributes to genomes diversification, speciation and finally to increase in biodiversity [8,35,83,96]. In mammals, differences in copy number between 5S and 45 rDNAs has been proved to be involved in pathological conditions. 

## 10. Conclusions

Nucleolus and rDNAs are the hub integrating environmental and intracellular signals [81] and the cellular stress sensor [153]. Moreover, rDNA copy number has been shown to play a crucial role in maintenance of the genome integrity and onset of diseases and senescence [162]. Therefore, the integrative cytogenomic analysis of not only fish rDNAomes represents another, so far unexploited, dimension of the genomics in fish *sensu* [73] and allows alternative insights into the complex interactions between cells and organisms and their environment. In future, more systematic studies on molecular cytogenetic detection of 5S and 45S rDNA in different populations and/or species will enable us to assess the role of rDNA spreading across chromosomes in genome differentiation at different environmental conditions. As we showed, the vertebrates’ rDNAome still represents a largely underestimated and unexploited genomic fraction with the huge potential to elucidate and proper understand crucial genomic functions and above all genome’s interactions with the environment.

## Figures and Tables

**Table 1:** Main cytogenomic traits in fish-like chordates.

Group	2*n* Chromosome Counts (Basic Features)	Micro- Chromosomes	CG Heterogeneity	WGD after the First Two Basal Vertebrates´ WGDs	C-value/Haploid DNA Content (pg) [12]	Specific Features in the Genome History and Chromosomal Evolution
Myxiniformes (hagfishes)	14–48	NO	unknown	not observed	Myxinidae 2.5–4.59	chromatin diminution, programmed genome rearrengement [13,14]
Petromyzontiformes (lampreys)	76–178	NO	GC-rich DNA repeats	not observed	Petromyzontidae 1.29–2.5	programmed genome rearrengement [14]
Chondrichthyes (cartilaginous fishes)	54–102	YES	observed, presumably satellite DNA	not observed	Chimeriformes 1.5-2 Selachimorpha ~3–17 Rajimorphii 2.7–17	AT/GC heterogeneity positively correlated with genome size [15]
Ceratodontiformes (lungfishes)	34–68	Only *N. forsteri* [15] otherwise not	unknown	not observed	*Neoceradotus* 52.75–74.86 *Lepidosiren* 80.55–123.9 *Protopterus* 40–132.8	“genomic obesity” without WGD documented [16,17]
Coelacanthiformes (lobe-finned fishes)	48	YES	unknown	not observed	*Latimeria* 2.8–6.6	chromosomes similar to ancient frogs [18]
Acipenseriformes (sturgeons, paddlefish)	~ 120–240–360	YES ~ 50%	NORs and GC-rich microchromosomes [7] andG-banding [19,20]	multiple in sturgeons, one in paddlefish	*Acipenser* 1.8–9.3 *Polyodon* 1.6–2.4	multiple WGD, ploidy diversity
Lepisosteiformes (gars)	56–58	small sized chromosomes	in both genera	not observed	*Atractosteus* 1.2 *Lepisosteus* 1.4	regionally high recombination rate
Amiiformes (bowfin)	46	NO	only NORs	not observed	*Amia calva* 1.2	convergent evolution with teleosts?
Polypteriformes (bichirs)	36–38 biarmed, extremelly large	NO	only NORs	not observed	*Erpetoichthys* 4.5 *Polypterus* 3.6-7.2	not investigated
Teleostei	~ 50 (exceptions up to 100–150 or more)	Micro B-chromosomes	only NORs	TGD and lineage specific WGDs	mostly 0.4- ~ 1.0	from genome compaction to lineage specific WGD

WGD: Whole-genome duplication; NOR: Nucleolar organizer region; pg: picograms.

^a^ orders and families mostly based on [22]; ^b^ c-value, based on [12] database; ^c^ *n*: based on genomic/sequence data, originates from NCBI, 2*n* based on cytogenetic data [86]; ^d^ number of assemblies currently released and level of assembly (C = contig, D = draft, S = scaffold, Ch = chromosomal level); ^e^ Sequencing methods (I = Illumina, PB = PacBio, S = Sanger), linkage map (LM) available.

## Appendix A. List of Fish Species with Sequenced Genomes Currently Available

**Table A1:** Detailed overview of fish species with a sequenced genome. Diverse levels of genome assemblies (draft, contig, scaffold, fully assembled genomes to the chromosome level) and numbers of assembly versions are listed together with basic cytogenetic traits (2*n*, C-value, GC%).

Species	Order ^a^	Family ^a^	Size (Mb)	C-val ^b^	*n*/2*n * ^c^	Assembly/Type ^d^	GC%	Notes ^e^
*Acanthochromis polyacanthus*	Ovalentaria	Pomacentridae	991.585	0.94	—	1/S	41.5	
*Amphilophus citrinellus*	Cichliformes	Cichlasomat., Heroini	844.903	—	—/48	1/S	41.4	
*Anguilla anguilla*	Anguilliformes	Anguillidae	1018.7	1.11–1.67	—/38	1/S	42.9	
*Anguilla japonica*	Anguilliformes	Anguillidae	1288.6	1.09	—/38	1/S	38.8	
*Anguilla rostrata*	Anguilliformes	Anguillidae	1413.03	1.01–1.66	—/38	1/S	41.0	
*Anoplopoma fimbria*	Perciform × Scorpaeniform	Anoplomatidae	699.326	0.71–0.84	—	1/C	40.3	
*Astyanax mexicanus*	Characiformes	Characidae	1335.24	—	25/50	1/Ch	38.4	
*Austrofundulus limnaeus*	Cyprinodontiformes	Rivulidae	866.963	—	—	1/S	41.1	
*Boleophthalmus pectinirostris*	Gobiiformes	Gobiid., Oxudercinae	955.752	—	—/46	1/S	40.1	
*Branchiostoma belcheri*	Cephalochordata	Branchiostomidae	426.124	—	—/36	2/S	40.15	
*Branchiostoma floridae*	Cephalochordata	Branchiostomidae	521.895	—	—	1/S	41.8	
*Callorhinchus milii*	Chimaeriformes	Callorhinchidae	974.499	1.94	—	1/S	42.6	
*Channa argus*	Perciformes	Channidae	615.3	0.63–0.77	—/48	1/D	—	
*Clupea harengus*	Clupeiformes	Clupeidae	807.712	—	—/50-52	1/S	44.5	
*Cottus rhenanus*	Perciformes	Cottidae	563.609	—	—	1/S	36.8	
*Cynoglossus semilaevis*	Pleuronectiformes	Cynoglossidae	470.199	0.62 *C. bilineatus*	22	1/Ch	41.27	
*Cyprinodon nevadensis*	Cyprinodontiformes	Cyprinodontidae	1011.85	—	—/48	1/S	39.0	
*Cyprinodon variegatus*	Cyprinodontiformes	Cyprinodontidae	1035.18	1.6	—/48	1/S	39.5	
*Cyprinus carpio*	Cypriniformes	Cyprinidae	1713.66	1.6–2.0	50/100	2/S, Ch	med 37.1	
*Danio rerio*	Cypriniformes	Cyprinidae	1679.2	1.6–2.3	25/48	4/3S, 1 Chr	med 36.7	
*Dicentrarchus labrax*	Moroniformes	Moronidae	675.917	0.78	—	2/S	40.4	
*Eptatretus burgeri*	Myxiniformes	Myxinidae/Eptatretinae	2608.38	2.98	/36	1/S	—	
*Esox lucius*	Esociformes	Esocidae	904.497	0.8–1.4	25/50	3/Ch	42.2	PB
*Fundulus heteroclitus*	Cyprinodontiformes	Fundulidae	1021.9	1.3–1.5	-/48	1/S	41.2	
*Gadus morhua*	Gadiformes	Gadidae	824.311	0.4–0.9	-/46	2/S	46.3	454, I, PB [124]
*Gasterosteus aculeatus*	Perciformes	Cottiodei, Gasterosteid.	446.611	0.6–0.7	—/42	2/S, Ch	44.6	LM [163]
*Haplochromis burtoni*	Perciformes × Cichliformes	Cichlidae (Afr.)	831.412	0.97	—/40	1/S	41.9	
*Hippocampus comes*	Syngnathiformes	Syngnathidae	493.776	—	—	1/S	43.7	
*Ictalurus punctatus*	Siluriformes	Ictaluridae	783.275	1.0	29/58	1/Ch	39.8	PB, I [164,165]
*Kryptolebias marmoratus*	Cyprinodontiformes	Rivulidae	680.367	—	—/48	2/S	med 39.5	
*Labeotropheus fuelleborni*	Perciformes × Cichliformes	Cichlidae (Afr.)	70.8584	—	—/44	1/S	42.1	
*Labrus bergylta*	Labriformes	Labridae	805.481	—	—	1/S	40.9	
*Larimichthys crocea*	Acanthuriformes	Sciaenidae	678.938	—	—	2/S	med 41.4	
*Lates calcarifer*	Perciformes	Centropomidae	668.481	0.7	—/48	2/S	med 40.6	
*Latimeria chalumnae*	Ceolacanthiformes	Coelacanthidae	2860.59	2.8–6.6	—/48	2/S	med 42.5	
*Lepisosteus oculatus*	Lepisosteiformes	Lepisosteidae	945.878	1.4	29/58	1/Ch	40.4	
*Lethenteron camtschaticum*	Petromyzontiformes	Petromyzontidae	1030.66	~1.4	—/144-162	1/S	48.1	
*Leuciscus waleckii*	Cypriniformes	Cyprinidae	752.539	congeners 1.2–1.5	—/50	1/S	37.4	
*Leucoraja erinacea*	Rajiformes	Rajidae	1555.46	3.5–4.6	—	1/C	40.3	
*Maccullochella peelii*	Perciformes ×	Percichthyidae	633.241	0.83	—	1/S	—	
*Maylandia zebra*	Perciformes × Cichliformes	Cichlidae (Afr.)	859.842	—	—	2/S	41.4	
*Mchenga conophoros*	Perciformes × Cichliformes	Cichlidae (Afr.)	73.4256	—	—	1/S	41.8	
*Megalobrama amblycephala*	Cypriniformes	Cyprinidae, Cultrinae	1116.0	1.12–1.35	—	1/D	37.3	[166]
*Melanochromis auratus*	Perciformes × Cichliformes	Cichlidae (Afr.)	68.2386	—	—/46	1/S	41.5	
*Miichthys miiuy*	Acanthuriformes	Sciaenidae	619.301	—	—	1/S	39.3	
*Mola mola*	Tetraodontiformes	Molidae	639.452	0.8–0.9	—/46	1/S	41.2	[167]
*Monopterus albus*	Synbranchiformes	Synbranchidae	684.144	0.6–0.9	—/24	1/S	41.5	
*Morone saxatilis*	Moroniformes	Moronidae	585.167	0.9	—/48	1/S	40.0	
*Neolamprologus brichardi*	Perciformes × Cichliformes	Cichlidae (Afr.)	847.91	—	-	1/S	42	
*Nothobranchius furzeri*	Cyprinodontiformes	Notobranchiidae	1132.74	1.56	19/38	4/S, Chr	43, 8	
*Nothobranchius kuhntae*	Cyprinodontiformes	Notobranchiidae	5.23461	—	—/38	1/S	44.8	
*Notothenia coriiceps*	Perciformes	Nototheniidae	636.614	—	—	1/S	40.8	
*Oncorhynchus kisutch*	Salmoniformes	Salmonidae	2369.93	2.6–3.0	30/60	1/Ch	43.6	
*Oncorhynchus mykiss*	Salmoniformes	Salmonidae	2179	1.9–2.9	29/60	2/Ch	med 43.7	
*Oreochromis niloticus*	Perciformes × Cichliformes	Cichlidae (Afr.)	1009.86	0.9–1.2	23/44	2/Ch	med 39.9	PB, I [168,169]
*Oryzias latipes*	Beloniformes	Adrianichthyidae	869.818	0.9–1.1	24/48	5/Ch	med 40.8	
*Pampus argenteus*	Scombriformes	Stromateidae	350.449	—	—	1/S	38.2	
*Paralichthys olivaceus*	Pleuronectiformes	Paralichthyidae	643.911	0.7	24/46–48	2/S, Ch	med 42.4	
*Periophthalmodon schlosseri*	Gobiiformes	Gobiid., Oxudercinae	679.761	0.96	—	1/S	40.2	
*Periophthalmus magnuspinnatus*	Gobiiformes	Gobiid., Oxudercinae	701.697	—	—	1/S	40	
*Petromyzon marinus*	Petromyzontiformes	Petromyzontidae	885.535	1.6–2.4	—/168	1/S	46.8	
*Pimephales promelas*	Cypriniformes	Cyprinidae	1219.33	1.1	—/50	2/S	med 40.6	
*Poecilia formosa*	Cyprinodontiformes	Poeciliidae	748.923	0.75–0.97	—/46	1/S	39.6	
*Poecilia latipinna*	Cyprinodontiformes	Poeciliidae	815.145	0.9–1.0	—/46	1/S	40.8	
*Poecilia mexicana*	Cyprinodontiformes	Poeciliidae	801.711	0.7–1.38	—/46	1/S	40.7	
*Poecilia reticulata*	Cyprinodontiformes	Poeciliidae	731.622	0.77–1.0	23/46	1/Ch	40.3	
*Protosalanx hyalocranius*	Osmeriformes	Salangidae	525	—	—	1/D	—	
*Pseudopleuronectes yokohamae*	Pleuronectiformes	Pleuronectidae	547.831	0.67	—/48	1/C	42	
*Pundamilia nyererei*	Perciformes × Cichliformes	Cichlidae (Afr.)	830.133	—	—	1/S	41.9	
*Pygocentrus nattereri*	Characiformes	Serrasalmidae	1285.35	—	—	1/S	40.6	
*Rhamphochromis esox*	Perciformes × Cichliformes	Cichlidae (Afr.)	71.2951	—	—	1/S	42.3	
*Rhincodon typus*	Orectolobiformes	Rhincodontidae	2931.6	—	—	1/S	41.8	
*Salmo salar*	Salmoniformes	Salmonidae	2966.89	3.0–3.3	29/60	1/Ch	43.9	PB, I, S [170]
*Scartelaos histophorus*	Gobiiformes	Gobiid., Oxudercinae	695.009	—	—	1/S	39.1	
*Scleropages formosus*	Osteoglossiformes	Osteoglossidae	777.359	—	—	4/S, Chr	med 43.9	
*Sebastes aleutianus*	Scorpaeniformes	Sebastidae	899.65	—	—	1/S	40.9	
*Sebastes minor*	Scorpaeniformes	Sebastidae	681.653	—	—	1/S	40.8	
*Sebastes nigrocinctus*	Scorpaeniformes	Sebastidae	746.045	—	—	1/S	40.8	
*Sebastes rubrivinctus*	Scorpaeniformes	Sebastidae	756.297	—	—	1/S	40.7	
*Sebastes steindachneri*	Scorpaeniformes	Sebastidae	648.011	—	—	1/S	40.7	
*Seriola dumerili*	Carangiformes	Carangidae	677.67	0.74	—	1/S	40.9	
*Seriola lalandi*	Carangiformes	Carangidae	685	—	—	1/S	-	PB, I
*Seriola quinqueradiata*	Carangiformes	Carangidae	med 750.45	0.83	—	2/S	40.25	
*Sinocyclocheilus anshuiensis*	Cypriniformes	Cyprinidae	1632.72	—	—	1/S	38	
*Sinocyclocheilus grahami*	Cypriniformes	Cyprinidae	1750.29	2.35	—/96	1/S	38.7	
*Sinocyclocheilus rhinocerous*	Cypriniformes	Cyprinidae	1655.79	—	—	1/S	38.1	
*Squalius pyrenaicus*	Cypriniformes	Cyprinidae	48.1393	2.4	—/50	1/C	51.8	
*Stegastes partitus*	Ovalentaria	Pomacentridae	800.492	—	—	1/S	42.1	
*Takifugu flavidus*	Tetraodontiformes	Tetraodontidae	378.032	—	—	1/S	45.6	
*Takifugu rubripes*	Tetraodontiformes	Tetraodontidae	391.485	0.4	22/44	1/Ch	45.8	
*Tetraodon nigroviridis*	Tetraodontiformes	Tetraodontidae	342.403	0.35–0.51	21/42	7/S, Ch	46.6	Genoscope
*Thunnus orientalis*	Scombriformes	Scombridae	684.497	—	—/48	1/C	39.7	
*Xiphophorus couchianus*	Cyprinodontiformes	Poeciliidae	708.396	0.75	—	1/S	40.9	
*Xiphophorus hellerii*	Cyprinodontiformes	Poeciliidae	733.802	0.7–1.0	—/48	1/S	41.2	
*Xiphophorus maculatus*	Cyprinodontiformes	Poeciliidae	729.664	0.8–1.0	—/48	1/S	39.8	

^a^ orders and families mostly based on [22]; ^b^ c-value, based on [12] database; ^c^ *n*: based on genomic/sequence data, originates from NCBI, 2*n* based on cytogenetic data [86]; ^d^ number of assemblies currently released and level of assembly (C = contig, D = draft, S = scaffold, Ch = chromosomal level); ^e^ Sequencing methods (I = Illumina, PB = PacBio, S = Sanger), linkage map (LM) available.
